# Design of a Vitronectin-Based Recombinant Protein as a Defined Substrate for Differentiation of Human Pluripotent Stem Cells into Hepatocyte-Like Cells

**DOI:** 10.1371/journal.pone.0136350

**Published:** 2015-08-26

**Authors:** Masato Nagaoka, Motohiro Kobayashi, Chie Kawai, Sunil K. Mallanna, Stephen A. Duncan

**Affiliations:** 1 Tenure-track Program for Innovative Research, University of Fukui, Yoshida-gun, Fukui, Japan; 2 Department of Cell Biology, Neurobiology and Anatomy, Medical College of Wisconsin, Milwaukee, Wisconsin, United States of America; 3 Division of Tumor Pathology, Department of Pathological Sciences, Faculty of Medical Sciences, University of Fukui, Yoshida-gun, Fukui, Japan; University of Minnesota Medical School, UNITED STATES

## Abstract

Maintenance and differentiation of human pluripotent stem cells (hPSCs) usually requires culture on a substrate for cell adhesion. A commonly used substratum is Matrigel purified from Engelbreth—Holm—Swarm sarcoma cells, and consists of a complex mixture of extracellular matrix proteins, proteoglycans, and growth factors. Several studies have successfully induced differentiation of hepatocyte-like cells from hPSCs. However, most of these studies have used Matrigel as a cell adhesion substrate, which is not a defined culture condition. In an attempt to generate a substratum that supports undifferentiated properties and differentiation into hepatic lineage cells, we designed novel substrates consisting of vitronectin fragments fused to the IgG Fc domain. hPSCs adhered to these substrates via interactions between integrins and the RGD (Arg-Gly-Asp) motif, and the cells maintained their undifferentiated phenotypes. Using a previously established differentiation protocol, hPSCs were efficiently differentiated into mesendodermal and hepatic lineage cells on a vitronectin fragment-containing substrate. We found that full-length vitronectin did not support stable cell adhesion during the specification stage. Furthermore, the vitronectin fragment with the minimal RGD-containing domain was sufficient for differentiation of human induced pluripotent stem cells into hepatic lineage cells under completely defined conditions that facilitate the clinical application of cells differentiated from hPSCs.

## Introduction

The generation of mature hepatocytes from hPSCs is a useful approach for therapeutic applications, researching drug metabolism, and the study of genetic diseases using patient-derived induced pluripotent stem (iPS) cells. Several studies have demonstrated induction of hepatic lineage cells from hPSCs *in vitro* [1–4], which have mostly used Matrigel as a substrate for cell adhesion. Matrigel is a gel matrix purified from Engelbreth—Holm—Swarm sarcoma cells, which consists of a mixture of extracellular matrix proteins, proteoglycans, and growth factors [5–7]. Because of the enriched basement membrane proteins and growth factors in Matrigel, it is used to induce differentiation, facilitate invasion of tumor cells, and support duct formation of epithelial cells as well as angiogenesis *in vitro*. Matrigel is also used for maintenance of rodent and human primary hepatocytes in culture [8–11]. Although it is a viable substitute for basement membrane, Matrigel is a relatively impure preparation with significant lot-to-lot variability in the content of growth factors and matrix proteins that can affect the reproducibility of cell differentiation. Another concern is the possibility for contamination by pathogens, which raises serious safety issues for clinical approaches using stem cell—derived cells and may affect functional studies of these cells.

For the maintenance of hPSCs, many substrates have been suggested as a substitute for Matrigel, including extracellular matrix proteins such as vitronectin [12], fibronectin [13,14], collagen [15], and laminin-511 [16,17], synthetic polymers and peptides [18–21], and recombinant proteins [22]. We have previously reported that a fusion protein of E-cadherin and the IgG Fc region supports the undifferentiated state of mouse and human PSCs [22,23]. Therefore, we used the same approach to develop a novel Fc fusion protein that could support the differentiation of hPSCs into hepatic lineage cells under defined conditions to eliminate xenogeneic components.

Vitronectin is an extracellular matrix protein consisting of somatomedin B, hemopexin and heparin-binding domains, and a vitronectin-coated surface can be used to maintain the pluripotency of hPSCs [12]. In addition, Chen et al. reported that a vitronectin fragment containing the RGD motif and hemopexin domain is sufficient to maintain and establish human iPS cells [24]. A surface coated with a synthetic RGD motif or heparin-binding peptide can also support the adhesion and pluripotency of hPSCs [18]. Furthermore, it has been reported that definitive endoderm cells differentiated from human embryonic stem (ES) cells express integrin alpha-v/beta-5, a receptor of vitronectin [25]. A surface coated with both fibronectin and vitronectin efficiently promotes the differentiation of human ES cells into definitive endoderm lineages [26]. In this study, we constructed novel substrates consisting of vitronectin fragments and IgG Fc fusion proteins, and examined whether these fusion proteins could support the maintenance and differentiation of hPSCs into hepatocyte-like cells under completely defined culture conditions.

## Materials and Methods

### Cell culture

Human ES cells (H9: WA09) and human iPS cells (K3 [27]) were maintained on a human E-cad-Fc fusion protein [22] in mTeSR1 medium (StemCell Technologies, Vancouver, BC) in a humidified atmosphere with 5% CO_2_/4% O_2_ at 37°C. The cells were passaged with enzyme-free Cell Dissociation Buffer (phosphate-buffered saline [PBS]-based; Life Technologies, Carlsbad, CA) before confluency by incubation at room temperature until the cells detached from the surface. The cells were then collected by centrifugation at 200 × *g* for 5 min at room temperature. All media contained 100 U/ml penicillin and 100 μg/ml streptomycin (Millipore, Billerica, MA). Cells cultured on vitronectin variants were treated with Accutase (Millipore) and passaged before confluency. For teratoma formation assays, human iPS cell lines (253G1 [28], 454E2 [29] and TIG120-4f1 [30]) were cultured on R-Fc in mTeSR1 medium. Human iPS cell line 201B6 [31] was used for differentiation into hepatocyte-like cells.

### Construction and expression of fusion proteins

To construct expression vectors for vitronectin variant-IgG Fc fusion proteins, cDNAs encoding human vitronectin variants were amplified by PCR with PrimeSTAR HS DNA polymerase (TaKaRa Bio Inc., Otsu, Japan) from a plasmid containing full-length human vitronectin cDNA (PlasmID Repository, clone ID: HsCD00045411, Boston, MA). The specific primers used for amplification are listed in Table 1. PCR products were digested with PciI and NotI, and then purified. The cDNAs of vitronectin variants and a mutant mouse IgG1 Fc domain (T252M-T254S)[32], which has a high affinity for protein A, were ligated into a pET14b (Novagen, Darmstadt, Germany) that was digested with NcoI and XhoI (blunt) to generate the expression vector for vitronectin variant-Fc fusion proteins. The fusion proteins were expressed by the Rosetta-gami B (DE3) pLysS strain (Novagen). The cells were collected by centrifugation, and the cell pellet was resuspended in lysis buffer (50 mM Tris-HCl, 50 mM NaCl, 0.1% Triton X-100, and 0.5 mM EDTA, pH 8.0) containing Lysonase (Millipore) and incubated for 30 min at room temperature. The lysate was centrifuged at 13,000 × *g* for 30 min at 4°C, and the supernatant was loaded onto an rProtein A FF column (GE Healthcare Life Sciences, Pittsburgh, PA). The column was washed with 20 mM phosphate buffer (pH 7.0), and the bound proteins were eluted using 0.1 M sodium citrate buffer (pH 2.7) followed by neutralization with a 1/5 volume of 1 M Tris-HCl (pH 9.0). Eluates were dialyzed against PBS for 3 days.

**Table 1:** Primer pairs used for construction of hVTN variants-Fc. Underline indicates PciI and NotI recognition sites, and mutated sequence is shown in italic letters.

R-Fc	gatcACATGTtgactcgcggggatgtgttc	gatcGCGGCCGCTttcctcaggtttcagaacag
R(D6E)-Fc	gatcACATGTtgactcgcggg*GAA*gtgttc	gatcGCGGCCGCTttcctcaggtttcagaacag
NC-Fc	gatcACATGTtgactcgcggggatgtgttc	gatcGCGGCCGCTggatggccggcgggagttct
NC(D6E)-Fc	gatcACATGTtgactcgcggg*GAA*gtgttc	gatcGCGGCCGCTggatggccggcgggagttct

### Preparation of substrate-coated surfaces

The purified solutions of vitronectin variants (R-Fc and NC-Fc) or recombinant human vitronectin (kindly provided by Primorigen Biosciences Inc., Madison, WI) were directly added to untreated polystyrene plates to prepare surfaces coated with recombinant proteins. After 1 h of incubation at 37°C, the plates were washed once with PBS, and cells were then seeded. BD Matrigel hESC-qualified Matrix (BD Biosciences, Bedford, MA) was diluted with DMEM according to manufacturer’s instruction and added to tissue culture-treated plates followed by incubation at room temperature for 1 h.

### Adhesion assays

For the adhesion assay, cells were seeded into 96-well plates pre-coated with mouse IgG (Jackson Immunoresearch Laboratories, West Grove, PA), recombinant human vitronectin, vitronectin variants, or hE-cad-Fc. After 1 day of culture, the medium and non-adherent cells were removed, and the cells were washed with culture medium. Adherent cells were then stained with Cell Proliferation Reagent WST-1 (Roche Applied Science, Indianapolis, IN). After incubation for 2.5 h, absorbance at 450 nm was measured using a microplate reader (BioTek Instruments, Winooski, VT). For the inhibition assay, resuspended cells were incubated with 50 μg/ml cyclo(Arg-Gly-Asp-D-Phe-Val) (RGDfV) peptide (Millipore) at room temperature for 30 min and then seeded onto surfaces coated with the various substrate proteins.

### Differentiation of hPSCs into multi-lineage cells

Human iPS cells cultured on various substrates were differentiated into three germ layer-derived lineages as described previously with minor modifications [33]. Briefly, 201B6 cells maintained on R-Fc, NC-Fc, or Matrigel were treated with Accutase and were plated on the Matrigel-coated surface. Cells were treated with 1% DMSO for 1 day before differentiation, and differentiation was induced under the following conditions. Ectoderm: RPMI-1640 media supplemented with B27 (Life Technologies), 500 ng/ml Noggin (Peprotech, Rocky Hill, NJ), and 10 μM SB431542 (Cayman Chemical, Ann Arbor, MI) for 2 days. Mesoderm: RPMI-1640 media supplemented with B27 (without insulin) and 100 ng/ml Activin A (R&D Systems, Minneapolis, MN) for 1 day followed by 20 ng/ml bone morphogenetic factor 4 (BMP4; HumanZyme Inc., Chicago, IL) for 1 day. Endoderm: RPMI-1640 media containing B27 (without insulin), 20 ng/ml BMP4, 10 ng/ml basic fibroblast growth factor (bFGF; ReproCELL, Tokyo, Japan) and 100 ng/ml Activin A for 1 day followed by 100 ng/ml Activin A for 1 day.

### Differentiation of hPSCs into hepatic progenitor cells

Differentiation of hPSCs into hepatocyte-like cells was induced by a modified four-step induction method [4]. Briefly, suspended cells were seeded onto polystyrene plates coated with Matrigel, human recombinant vitronectin, R-Fc, or NC-Fc. Before confluency, the cells were treated with RPMI-1640 medium containing 100 ng/ml Activin A and B27 supplement (without insulin; Life Technologies) for 5 days in a normoxic atmosphere (20% O_2_). The cells were then specified into the hepatic lineage by treatment with RPMI-1640/B27 (with insulin) medium containing 10 ng/ml bFGF and 20 ng/ml BMP4 for 5 days in a hypoxic atmosphere (4% O_2_). After specification, the cells were cultured in RPMI-1640/B27 medium with 20 ng/ml hepatocyte growth factor for 5 days (4% O_2_) and then in HCM (without epidermal growth factor; Lonza, Basel, Switzerland) supplemented with 20 ng/ml oncostatin M for 5 days (20% O_2_).

### Immunocytochemistry

Cells were fixed with cold methanol for 20 min at −20°C. The fixed cells were incubated with 1% bovine serum albumin/PBS for 1 h at room temperature and then stained with primary antibodies for 2 h, followed by Alexa Fluor-conjugated secondary antibodies (Life Technologies) for 1 h. The following primary antibodies were used in this study: rabbit anti-Oct3/4 antibody (H-134; Santa Cruz Biotechnology, Santa Cruz, CA), rabbit anti-Nanog antibody (D73G4; Cell Signaling Technology, Danvers, MA), goat anti-GATA4 antibody (R&D Systems), goat anti-Sox17 antibody (R&D Systems), goat anti-Sox1 antibody (R&D Systems), goat anti-HNF4A antibody (C-19, Santa Cruz Biotechnology), goat anti-Brachyury antibody (C-19; Santa Cruz Biotechnology), mouse anti-alpha-fetoprotein (AFP) antibody (Sigma, St. Louis, MO), and rabbit anti-albumin (ALB) antibody (Dako, Glostrup, Denmark). Nuclei were counterstained with 0.5 μg/ml DAPI (Life Technologies). Samples were observed by fluorescence microscopy.

### Flow cytometric analysis

To analyze CXCR4 expression after differentiation, the cells were treated with Accutase and then stained with a phycoerythrin-conjugated anti-CXCR4 antibody (R&D Systems). Stained cells were analyzed using the Guava EasyCyte system (Millipore).

### Reverse transcription (RT)-PCR analysis

Total RNA was isolated with an RNeasy Plus Mini Kit (Qiagen, Valencia, CA). First-strand cDNA was synthesized from 200 ng total RNA using ReverTra Ace (TOYOBO Life Science, Osaka, Japan) and random primers (nonamers). PCR was carried out with Taq polymerase (New England Biolabs, Ipswich, MA). Total RNA from human adult liver samples (five-donor pool) and fetal liver samples (BioChain Institute Inc., Newark, CA) were used as positive controls. Oligonucleotide primers used in this study are listed in Table 2. Amplicons were analyzed by 2% agarose gel electrophoresis.

**Table 2:** Primer pairs for RT-PCR analysis.

Gene (Unigene Symbol)	Sequence	Reference
*POU5F1* (Hs.249184)	GACAGGGGGAGGGGAGGAGCTAGG	[31]
	CTTCCCTCCAACCAGTTGCCCCAAAC	
*NANOG* (Hs.635882)	CAGCCCCGATTCTTCCACCAGTCCC	[31]
	CGGAAGATTCCCAGTCGGGTTCACC	
*DLK1* (Hs.533717)	ACTGCCAGAAAAAGGACGGG	-
	GAAGTCGCCCCCAATGTCAG	
*AFP* (Hs.518808)	GAATGCTGCAAACTGACCACGCTGGAAC	[31]
	TGGCATTCAAGAGGGTTTTCAGTCTGGA	
*HNF4A* (Hs.116462)	CACGGGCAAACACTACGGT	[34]
	TTGACCTTCGAGTGCTGATCC	
*ALB* (Hs.418167)	GCCTGTTGCCAAAGCTCGAT	[35]
	GCGAGCTACTGCCCATGCTT	
*ASGR1* (Hs.12056)	TTGTGGTTGTCTGTGTGATCG	-
	GTCCTTTCTGAGCCATTGCC	
*TDO2* (Hs.183671)	CCAGGTGCCTTTTCAGTTGC	-
	CTTCGGTATCCAGTGTCGGG	
*GAPDH* (Hs.544577)	CGGATTTGGTCGTATTGGGC	-
	GACTCCACGACGTACTCAGC	

Expression levels of cadherin and matrix metalloproteinase (MMP) family proteins were measured by qPCR assay using THUNDERBIRD Probe qPCR Mix (TOYOBO Life Science) and PrimeTime qPCR Assays (Integrated DNA Technologies Inc., Coralville, IA). The following probe sets were used in this study: *ACTB* (Hs.PT.39a.22214847), *POU5F1* (Hs.PT.58.14494169.g), *MMP2* (Hs.PT.58.39114006), *MMP14* (Hs.PT.58.41036041), *CDH1* (Hs.PT.58.3324071), and *CDH2* (Hs.PT.58.22217374). The expression level of each gene was normalized with that of *ACTB*, and relative quantification was calculated using the ΔΔCT method against a day 0 sample (undifferentiated cells).

Differentiation to hepatocyte-like cells was verified by real-time quantitative PCR (qPCR) analysis using KOD SYBR qPCR Mix (TOYOBO Life Science) and PrimerArray Hepatic Differentiation (PH017; TaKaRa Bio Inc.). The expression level was normalized against *GAPDH*, and data was analyzed using the GeneSpring GX software (Agilent Technologies).

### Teratoma formation assay

Cells cultured in 6-well plates coated with R-Fc were collected by Accutase treatment and resuspended in 50 μl mTeSR1 medium. Then, the cells were mixed with the same volume of Matrigel and subcutaneously injected into immunocompromised SCID (C.B-17/Icr-*scid/scid*Jcl) mice (CLEA Japan, Inc., Tokyo, Japan). After 14 weeks, teratomas were surgically dissected from the mice. Samples were fixed in 10% formaldehyde neutral solution (Wako Pure Chemical, Osaka, Japan) and embedded in paraffin. Sections were processed for hematoxylin and eosin staining. All experimental procedures and protocols were reviewed and approved by the Animal Research Committee of the University of Fukui.

### Cellular uptake of indocyanine green (ICG) or human low-density lipoprotein (LDL)

Cells were incubated with 1mg/ml indocyanine green (Wako Pure Chemical) or 20 μg/ml DiI-LDL complex (Life Technologies) for 60 min at 37°C. Cells were then washed with PBS, and cellular uptake was observed under microscope.

### Statistical analysis

Values are presented as the means ± SD or SEM. Statistical significance was assessed using the paired Student’s *t*-test. The probability level accepted for significance was *P* < 0.05.

## Results

### Construction of vitronectin variants as defined substrates for hPSCs

**Fig 1:**
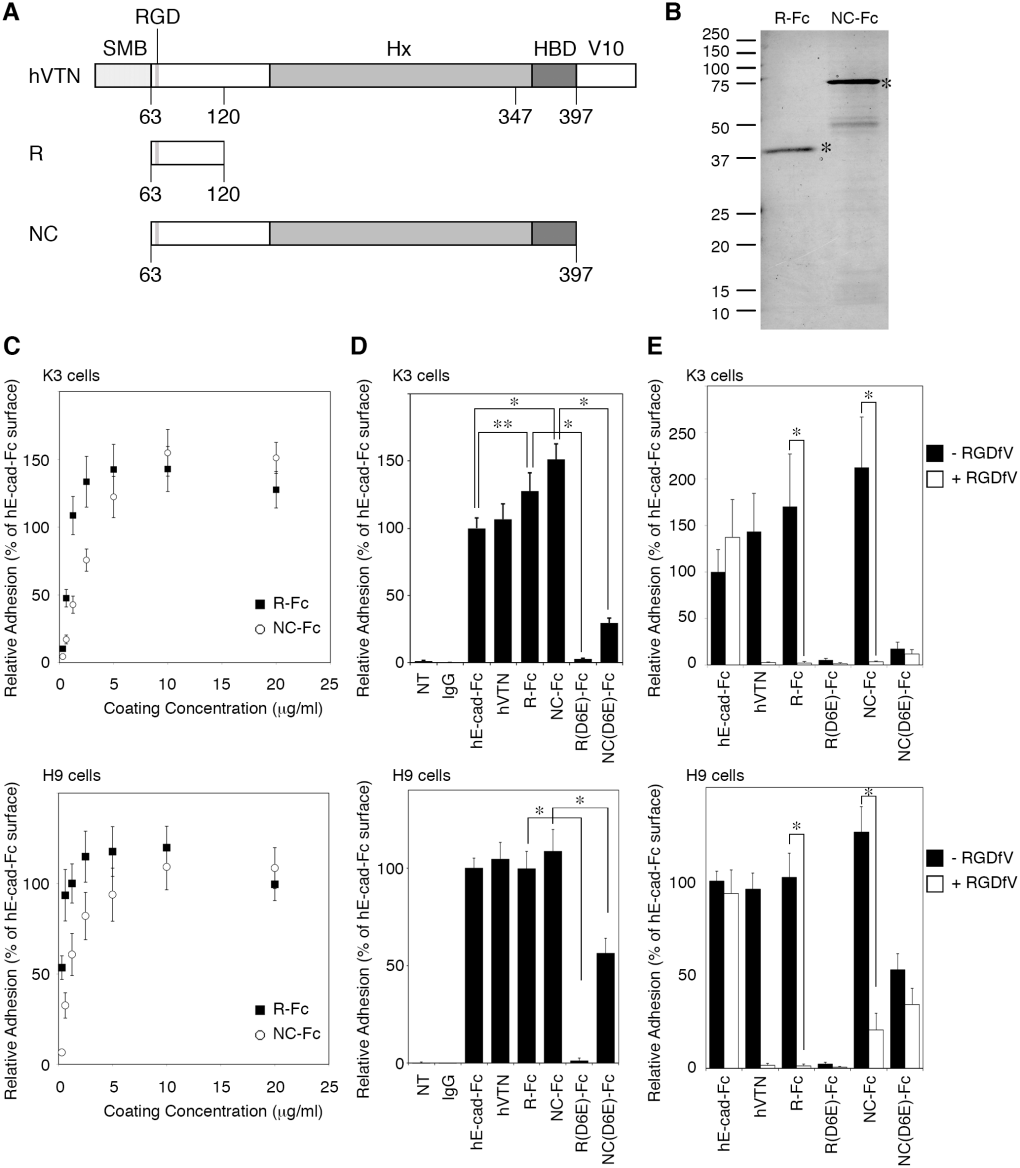
Construction of vitronectin variant-Fc fusion proteins and the adhesion properties of human pluripotent stem cells (hPSCs). (A) Schematic representation of the construction of vitronectin variants. All variants were fused with the mouse IgG_1_ Fc region. SMB, somatomedin B domain; Hx, hemopexin homology domain; HBD, heparin-binding domain; V10, vitronectin V10 subunit. (B) Purified vitronectin variant-Fc fusion proteins were separated by SDS-PAGE and stained with Coomassie Brilliant Blue. The estimated molecular weights were 32.6 and 64.6 kDa for R-Fc and NC-Fc, respectively. The purified proteins are marked with an asterisk. (C) Effect of the coating concentration of R-Fc and NC-Fc on hPSC adhesion. The number of adherent cells was estimated by a WST-1 assay and compared with the cell number on surfaces coated with 20 μg/ml hE-cad-Fc. Data are presented as the means ± SEM of five independent experiments. (D) The number of adherent cells on various substrates was estimated by the WST-1 assay. Each protein was coated at 20 μg/ml. Data are presented as the means ± SEM of five independent experiments. *p < 0.001, **p < 0.01. NT, non-treated polystyrene surface. (E) Inhibition of cell adhesion by the RGDfV peptide (50 μg/ml). K3 and H9 cells were treated with or without the RGDfV peptide for 30 min before seeding. The number of adherent cells was estimated by the WST-1 assay. Data are presented as the means ± SD of three independent experiments. *p < 0.001. hVTN indicates recombinant human vitronectin.

To establish a defined condition for maintenance and differentiation of hPSCs into the hepatic lineage, we tested two vitronectin variants fused with the Fc region of mouse IgG_1_ (Fig 1A). Because the N-terminal somatomedin B domain is not necessary for adhesion and maintenance of hPSCs [24], the somatomedin B domain was removed (R-Fc contained the RGD motif; NC-Fc consisted of RGD, hemopexin and heparin-binding domains, which is similar to VTN-NC reported by Chen et al. [24]). These fusion proteins were expressed in the Rosetta-gami B strain, and their purity was analyzed by SDS-PAGE followed by Coomassie Brilliant Blue staining (Fig 1B). The purity of R-Fc was more than 95%, and NC-Fc contained minor contaminating protein bands.

### Adhesion of hPSCs onto vitronectin variants

First, we examined the adhesion efficiency of hPSCs on surfaces coated with vitronectin variant-Fc fusion proteins. K3 human iPS cells and H9 human ES cells were harvested using an enzyme-free chelating buffer from an E-cad-Fc-coated surface and then plated onto various substrate-coated surfaces. hPSCs adhered to R-Fc and NC-Fc in a coating concentration-dependent manner with maximal adhesion observed at 5–10 μg/ml (Fig 1C). In contrast, hPSCs failed to adhere to either an uncoated surface or a surface coated with IgG. The adhesion efficiency of K3 cells on R-Fc and NC-Fc was better than that on the E-cad-Fc-coated surface, whereas no significant differences were found in the adhesion of H9 cells (Fig 1D). To assess whether adhesion of hPSCs onto vitronectin variants is mediated by an interaction with the RGD motif, we prepared RGE [36] mutants [R(D6E)-Fc and NC(D6E)-Fc] and analyzed the adhesion of hPSCs on these mutants. As shown in Fig 1D, the adhesion of hPSCs to vitronectin variant-Fc fusion proteins was completely disrupted by substitution of the Asp residue with the Glu residue within the RGD. Few cells adhered to the surface coated with vitronectin variants containing the heparin-binding domain [NC(D6E)-Fc], indicating that the heparin-binding domain is not sufficient for strong adhesion of hPSCs (Fig 1D). Furthermore, the adhesion of hPSCs to vitronectin variants was inhibited by pre-incubation with the RGDfV peptide, which is a blocking peptide for the RGD sequence, whereas no effect was observed on cell adhesion to the E-cad-Fc-coated surface (Fig 1E). These data indicate that R-Fc and NC-Fc support the adhesion of hPSCs via the RGD motif in the vitronectin sequence.

**Fig 2:**
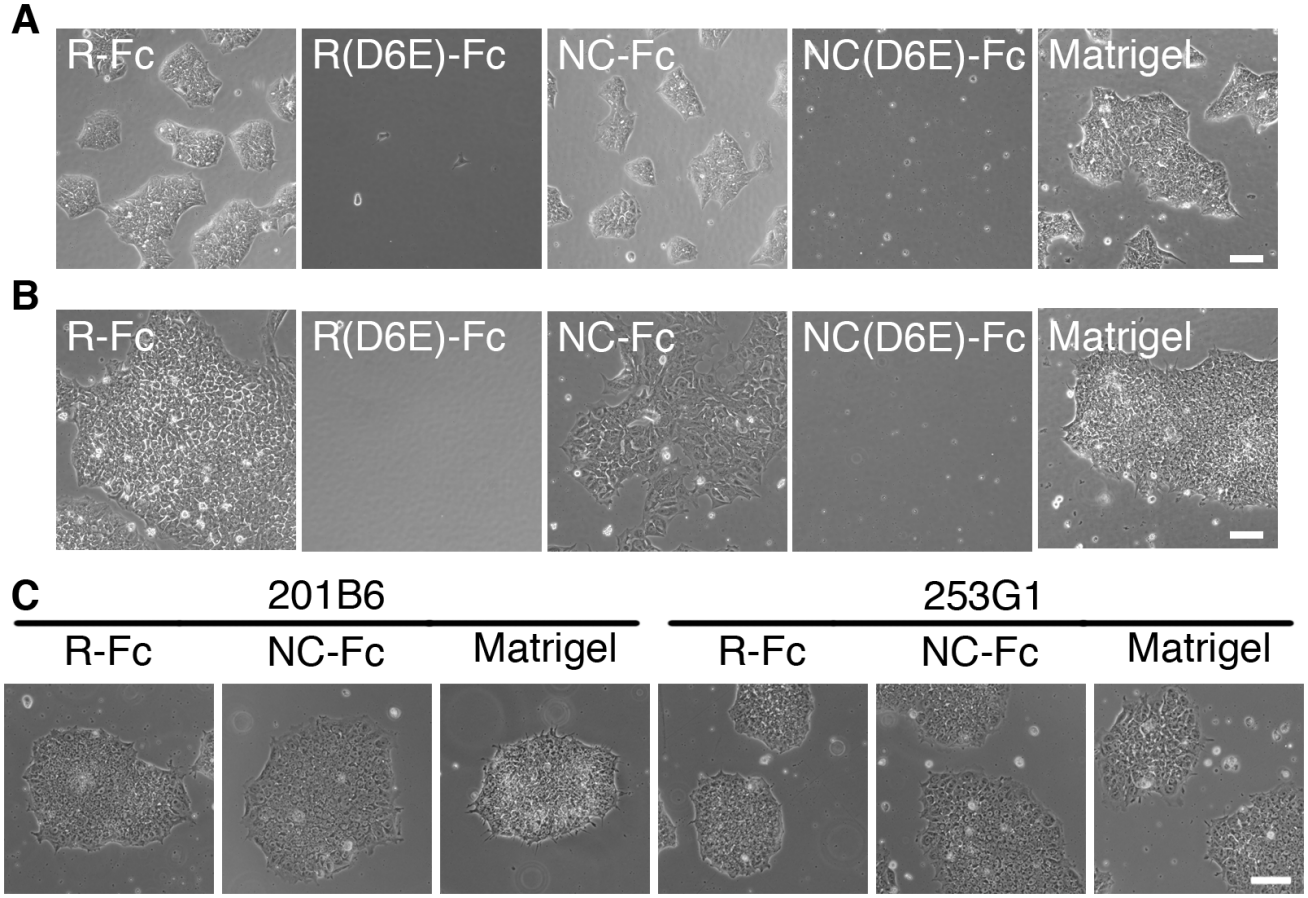
Morphology of hPSCs on vitronectin variant-coated surfaces. Phase contrast micrographs showing the morphology of H9 human ES cells (A) and K3 human iPS cells (B) cultured on surfaces coated with Matrigel, R-Fc, NC-Fc, or D6E mutants at 3 days after seeding. Scale bar indicates 100 μm. (C) Morphological observation of 201B6 and 253G1 hiPS cells that were maintained on Matrigel-, R-Fc- or NC-Fc-coated surface for 6 passages.

The morphology of hPSCs grown on R-Fc-coated surface was similar to that of hPSCs grown on Matrigel-coated surface (Fig 2). H9 hES cells and two hiPS cell lines (201B6 and 253G1) all exhibited similar morphology on R-Fc, NC-Fc, and Matrigel-coated surface (Fig 2A and 2C). However, the iPS cell line K3 adopted an epithelial-like morphology on the NC-Fc-coated surface (Fig 2B). This difference may be attributable to diversity amongst iPS cell lines.

### Maintenance of the undifferentiated state of hPSCs on recombinant vitronectin-coated surfaces

**Fig 3:**
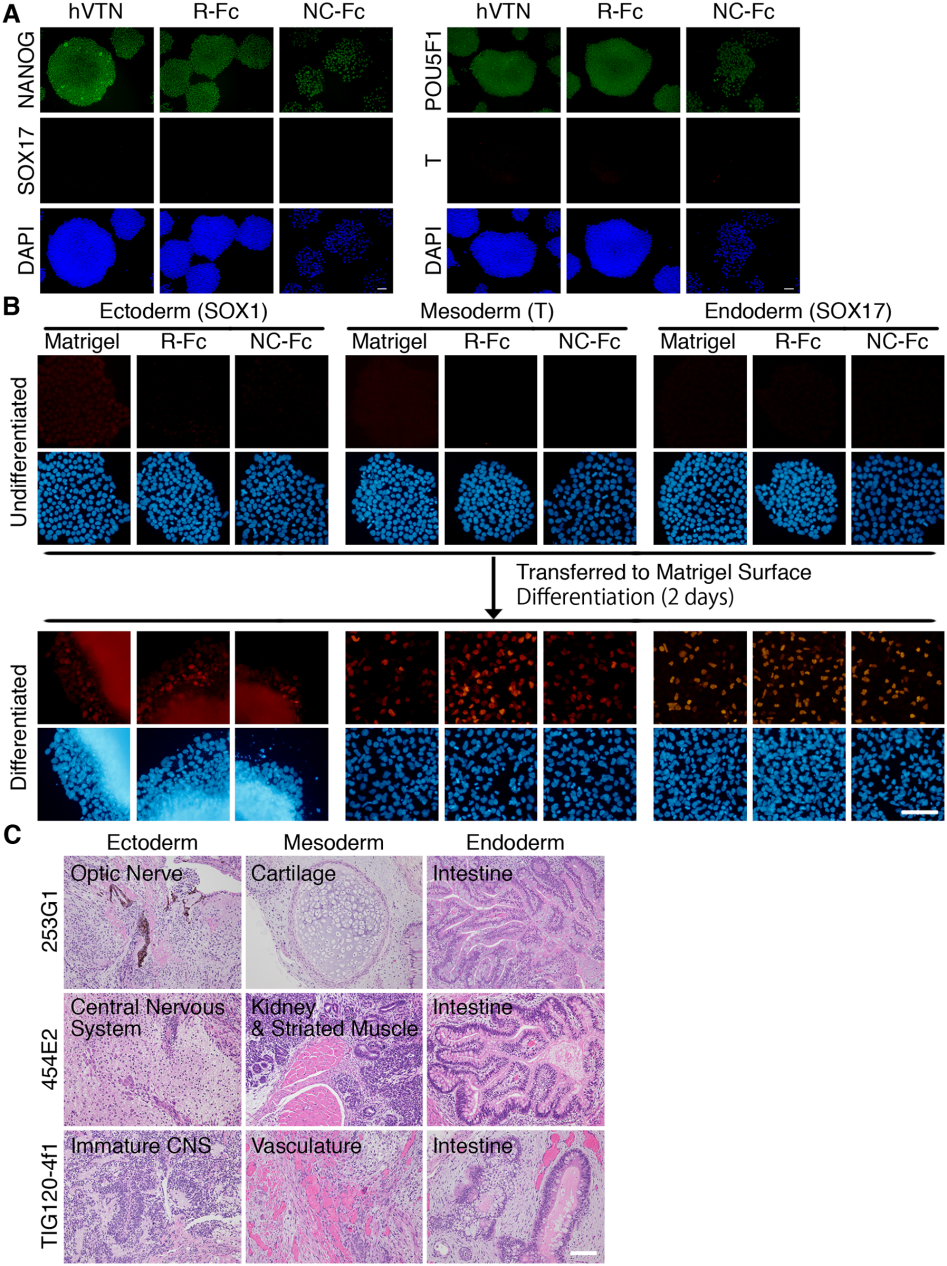
Maintenance of hPSCs on vitronectin variant-coated surfaces. (A) Immunocytochemistry of NANOG, SOX17, POU5F1, and T (Brachyury) in K3 hiPS cells cultured on recombinant human vitronectin (hVTN)-, R-Fc- or NC-Fc-coated dishes for 5 days. Nuclei were counterstained with DAPI. Scale bar indicates 100 μm. (B) Differentiation potential of hiPS cells cultured on R-Fc or NC-Fc into the ectodermal (SOX1), mesodermal (T), and endodermal (SOX17) lineages was examined. 201B6 cells were maintained on Matrigel, R-Fc or NC-Fc for 7 passages and transferred on Matrigel-coated surface to induce differentiation. (C) Characterization of teratomas formed by human iPS cells cultured on the R-Fc-coated surface. Teratomas generated from three different human iPS cell lines (253G1, 454E2, and TIG120-4f1) contained tissues derived from all three germ layers. Typical tissues are shown. Ectoderm: mature and immature central nervous systems (CNS), and optic nerve; Mesoderm: cartilage, kidney, striated muscle, and vasculature; Endoderm: intestine. Scale bar indicates 100 μm.

Because hPSCs could adhere and proliferate on vitronectin fragment-Fc fusion proteins, we determined whether R-Fc and NC-Fc supported the pluripotency of hPSCs. hPSCs cultured on R-Fc or NC-Fc maintained their expression of POU5F1 (OCT4) and NANOG, but no cells expressed SOX17 or T (Brachyury) (Fig 3A). Aditionally, hiPS cells cultured on R-Fc or NC-Fc maintained the ability to differentiate into ectoderm, mesoderm and endoderm lineage cells *in vitro* (Fig 3B). Next, we examined whether hPSCs could differentiate into lineages of all three germ layers after several passages on the R-Fc-coated surface. Three hiPS cell lines were maintained on R-Fc-coated surfaces in mTeSR1 medium for 33 (253G1 and 454E2) or 24 passages (TIG120-4f1), and then subcutaneously transplanted into immunocompromised SCID mice. At 14 weeks after transplantation, teratomas were generated, which contained tissues derived from all three germ layers (Fig 3C).

These results indicate that hPSCs can be maintained on surfaces coated with the R-Fc fusion protein that has a minimal integrin ligand.

### Differentiation of hPSCs on recombinant vitronectin-coated surfaces

**Fig 4:**
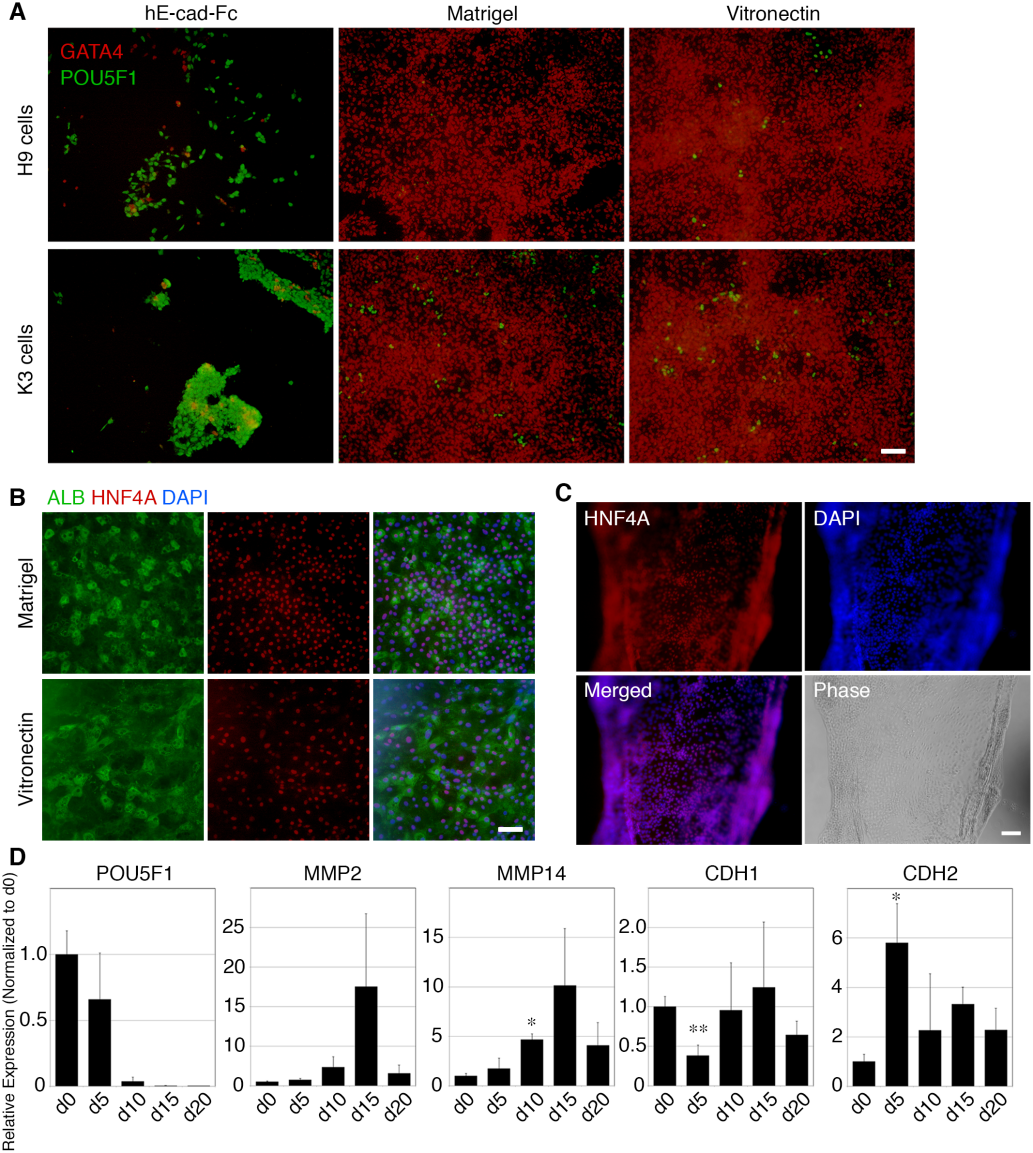
Differentiation of hPSCs into definitive endoderm cells on various substrates. (A) Most cells expressed the pluripotency marker POU5F1 on the hE-cad-Fc-coated surface, but almost all cells differentiated into definitive endoderm cells expressing GATA4 on vitronectin- and Matrigel-coated surfaces. (B) The vitronectin-coated surface supported differentiation of K3 cells into ALB^+^ hepatocyte-like cells at day 20 of differentiation. (C) Differentiating cells detached from the vitronectin-coated surface at day 10, although these cells expressed HNF4A. Scale bar indicates 100 μm. (D) Quantitative PCR analyses of cadherin and MMP gene expression during differentiation of human iPS cells. 201B6 cells were differentiated into hepatocyte-like cells on an R-Fc-coated surface, and total RNA was isolated at days 0, 5, 10, 15, and 20 of differentiation. Data are the means ± SD of three independent differentiation experiments. *p < 0.001, **p < 0.005 vs. day 0 (d0) sample.

First, we examined several recombinant proteins to determine whether they could support the differentiation of hPSCs into hepatic lineage cells as a substitute for Matrigel. Haque et al reported that mouse ES/iPS cells can be directly differentiated into hepatocyte lineage cells on an E-cad-Fc-coated surface [37]. However, hPSCs differentiated on an E-cad-Fc-coated surface still maintained expression of POU5F1 at the early stages of differentiation, and most GATA4-positive differentiated cells detached from the surface (Fig 4A). D’Amour et al. reported decreased E-cadherin expression and increased N-cadherin expression during differentiation of hPSCs into definitive endoderm cells [38]. Consistently, our quantitative PCR analysis revealed simultaneous down-regulation of *CDH1* (E-cadherin) and up-regulation of *CDH2* (N-cadherin) mRNA at day 5 of differentiation (Fig 4D). These findings indicate that an E-cad-Fc is unsuitable for use as a substrate for differentiation of hPSCs into definitive endoderm cells.

Definitive endoderm cells differentiated from hPSCs highly express a vitronectin receptor, integrin alpha-v/beta-5 [25], and we found that hPSCs were successfully differentiated into definitive endoderm cells on the full-length recombinant vitronectin-coated surface (Fig 4A). hPSCs were differentiated into ALB^+^/AFP^+^/HNF4A^+^ hepatocyte-like cells on the vitronectin-coated surface after 20 days of differentiation [4] (Fig 4B). However, in several culture wells the cells had detached as sheets from the surface during specification periods (days 7–10 of differentiation), although they still expressed HNF4A (Fig 4C).

Expression of MMP-2 and MMP-14 in the liver bud during mouse liver development promotes invasion of hepatoblasts into the septum transversum [39]. Vitronectin is considered as a putative substrate of MMP-2 [40]. Furthermore, MMP-14 activates MMP-2 by cleaving the MMP-2 pro-domain, and an activated mutant form of MMP-14 can directly cleave vitronectin [41]. Therefore, we surmise that MMP expression is induced during the specification stage of the differentiation protocol (day 5–10), and the expression of MMP results in degradation of vitronectin on the surface of the culture plate. We measured expression levels of *MMP2* and *MMP14* during differentiation of hiPS cells by qPCR. The expression of *MMP2* and *MMP14* was enhanced during day 5–10 of differentiation (Fig 4D), which is consistent with a previous report [35].

**Fig 5:**
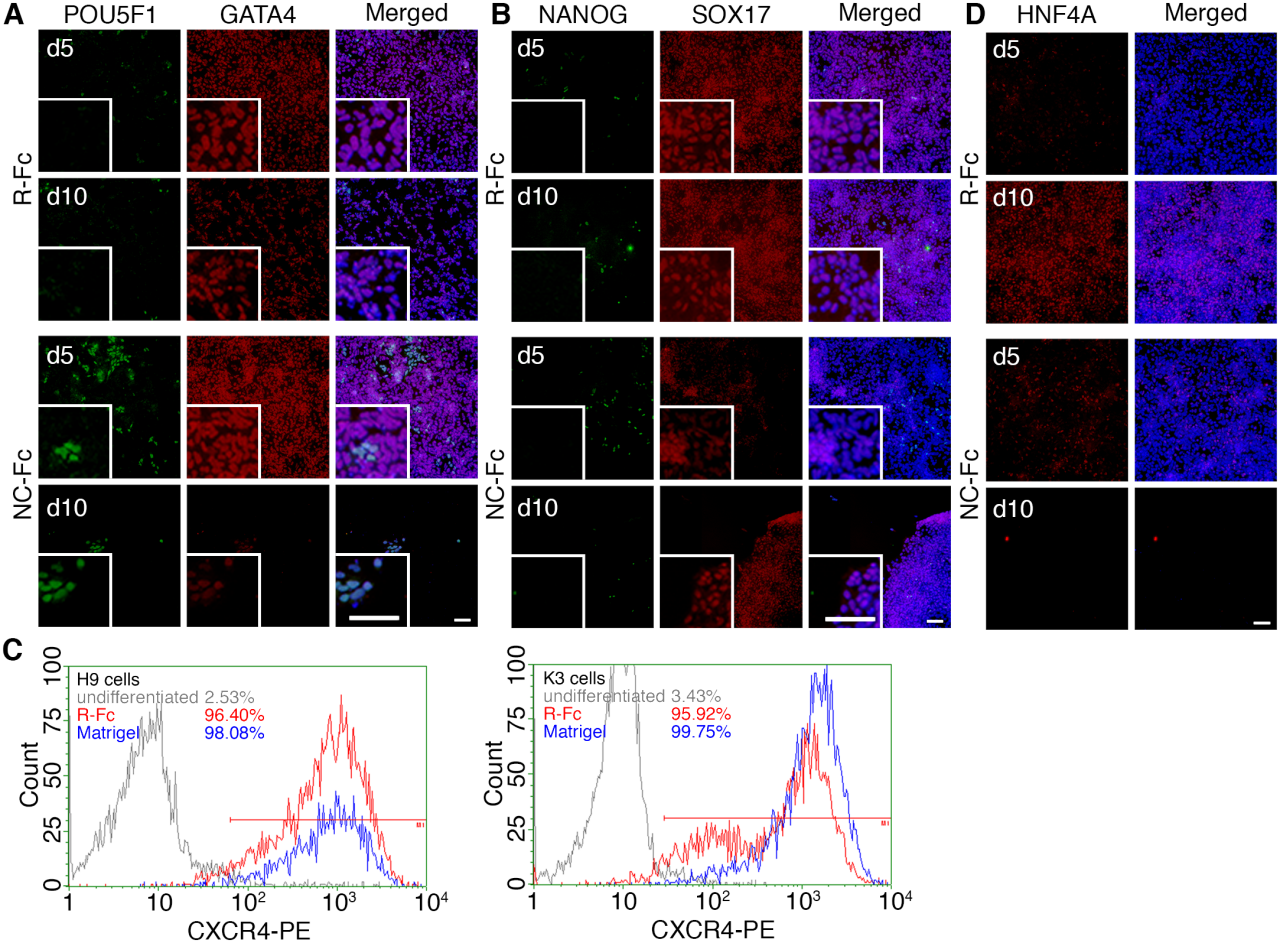
Differentiation of hPSCs on vitronectin variant-coated surfaces into hepatoblasts. (A and B) H9 human ES cells were induced to differentiate into the hepatic lineage on R-Fc- or NC-Fc-coated surfaces. The cells were fixed and stained at day 5 (definitive endoderm stage) and day 10 (specified hepatic lineages) to detect pluripotency markers (A: POU5F1; B: NANOG) and definitive endoderm markers (A: GATA4; B: SOX17). Inserted figures showed the magnified images. (C) H9 hES and K3 hiPS cells were induced to differentiate into definitive endoderm cells on Matrigel or R-Fc, and the expression of CXCR4 was analyzed by flow cytometry at day 5. Undifferentiated cells were used as a negative control. (D) Differentiated cells were stained for HNF4A (a marker of specified cells) at day 10. Scale bars indicate 100 μm.

Because full-length vitronectin did not appear to be a suitable substrate for the differentiation of hPSCs into hepatocyte lineages, we tested whether R-Fc and NC-Fc could support differentiation (Fig 5). After 5 days of treatment with Activin A, hPSCs differentiated into definitive endoderm cells expressing SOX17 and GATA4 on R-Fc and NC-Fc (Fig 5A and 5B). Furthermore, more than 90% of cells differentiated on the R-Fc-coated surface were positive for CXCR4 at day 5 of differentiation (Fig 5C), indicating that R-Fc may be a substitute for Matrigel. At day 10 of differentiation, most cells on R-Fc expressed HNF4A (Fig 5D), a marker of hepatic progenitor cells. However, similar to cells on the vitronectin-coated surface, the differentiated cells detached from the surface coated with NC-Fc at day 10 (Fig 4C). In contrast, when cells were induced to differentiate on R-Fc substrate they reproducibly remained attached throughout the entire process.

**Fig 6:**
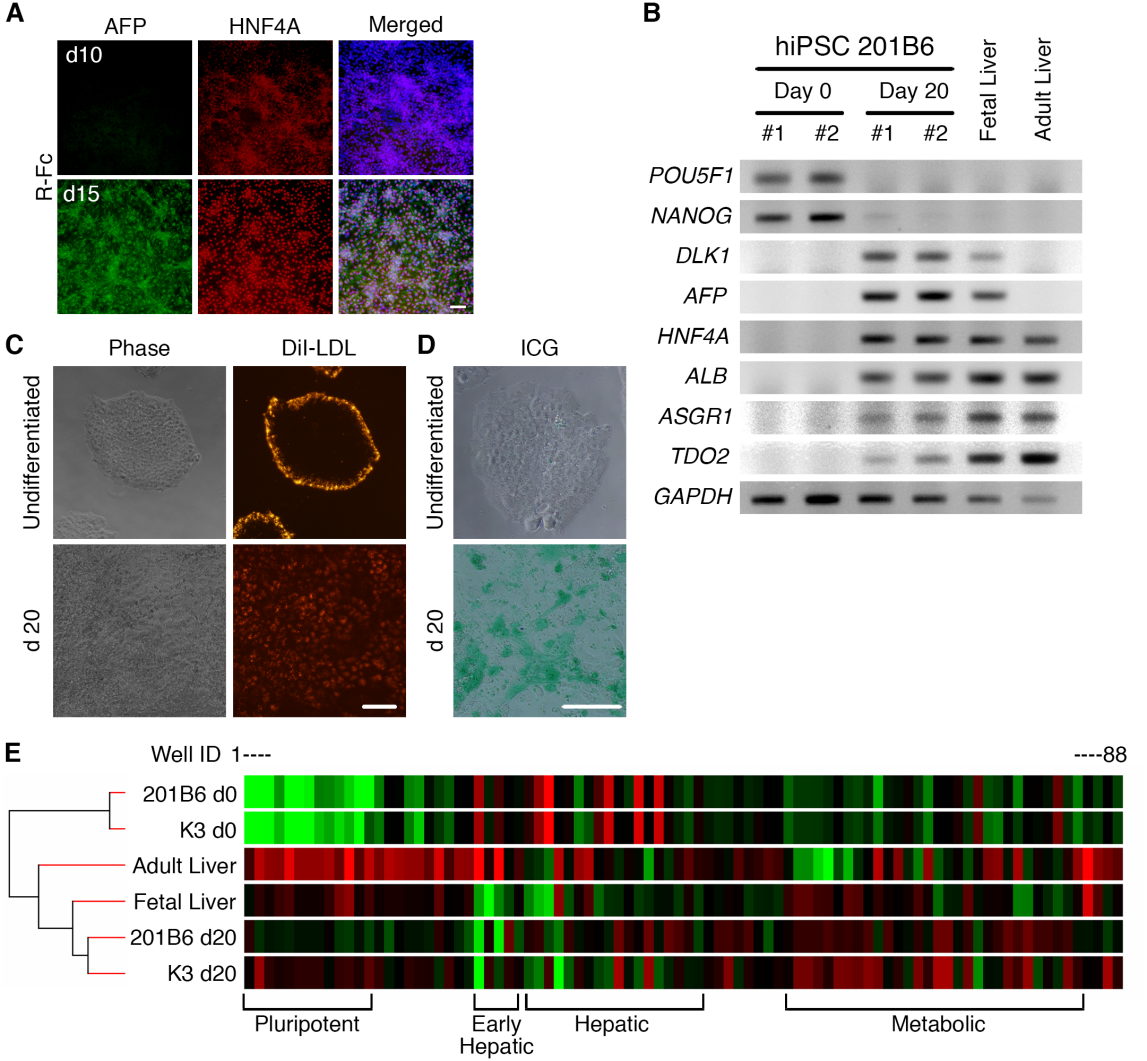
Differentiation of hPSCs on vitronectin variant-coated surfaces into hepatic lineage cells. (A) Hepatic progenitor cells were stained for AFP and HNF4A at days 10 and 15. In merged images, nuclei were counterstained with DAPI. (B) Expression of mRNAs for specific markers of undifferentiated cells (*POU5F1* and *NANOG*), hepatoblasts (*DLK1*, *AFP*, and *HNF4A*) and hepatocytes (*HNF4A*, *ALB*, *ASGR1*, and *TDO2*) was analyzed by RT-PCR. 201B6 human iPS cells were differentiated into hepatocyte-like cells, and total RNA was isolated from two independent differentiation experiments. Total RNAs from fetal and adult livers were used as controls for differentiated cells. (C and D) Cellular uptake of DiI-LDL (C) or ICG (D) by undifferentiated or differentiated (day 20) 201B6 iPS cells on R-Fc-coated surface. Scale bars indicate 100 μm. (E) Heatmap of qPCR analysis using PrimerArray assays and hierarchical clustering to compare expression levels of specific genes during hepatic differentiation. 201B6 cells were differentiated into hepatocyte-like cells on an R-Fc-coated surface (201B6 d20), and the expression levels of marker genes were compared with those of K3 human iPS cells differentiated on a Matrigel-coated surface (K3 d20). Well ID indicates 88 specific primer pairs of PrimerArray assays (PH017).

For further differentiation, the specified hepatic progenitor cells on the R-Fc-coated surface were treated with hepatocyte growth factor for an additional 5 days, and expression of AFP and HNF4A was detected by immunocytochemistry. Fig 6A shows that more than 90% of cells expressed HNF4A, and that HNF4A-positive cells co-expressed AFP at day 15. Human iPS cells cultured on the R-Fc-coated surface were efficiently differentiated into hepatocyte-like cells that expressed several markers of hepatic lineage cells after 20 days of differentiation (Fig 6B). The differentiated cells showed the ability to incorporate low-density lipoprotein (LDL), while undifferentiated human iPS cells showed non-specific accumulation of DiI-LDL complex at the peripheral region of the colonies (Fig 6C). Cellular uptake of indocyanine green (ICG) is also considered as a specific function of hepatocyte-like cells[42]. Cells differentiated on R-Fc-coated surface efficiently took up ICG after 1 h incubation (Fig 6D). These results indicate that human iPS cells could be differentiated into functional hepatocyte-like cells on R-Fc-coated surface. Furthermore, cells differentiated on an R-Fc-coated surface exhibited a similar gene expression profile to cells differentiated on a conventional Matrigel-coated surface. In addition, the gene expression profile of hiPS cell-derived hepatocyte-like cells was more akin to fetal liver cells rather than adult liver cells. This suggests that cells differentiated on both R-Fc and Matrigel were still immature hepatoblast-like cells (Fig 6E).

Cumulatively, these results demonstrate that all vitronectin variants tested are useful for the differentiation of hPSCs into definitive endoderm cells, but full-length vitronectin and NC-Fc could not support cell attachment during the specification of the endoderm to a hepatic fate. In contrast, when hPSCs were differentiated on the R-Fc-coated surface they remained attached throughout the differentiation process and were able to generate hepatocyte—like cells with high efficiency.

## Discussion

Vitronectin is a reliable substrate for the maintenance of hPSCs and differentiation of definitive endoderm as a substitute for Matrigel. However, the use of vitronectin for the differentiation into hepatic cells has not been described. Here, we showed that a small fragment of vitronectin is sufficient to maintain the pluripotency of hPSCs and facilitates differentiation of the cells toward hepatocytes. We also found that RGD-containing vitronectin variants (R-Fc and NC-Fc) supported efficient adhesion and propagation of hPSCs (Fig 1). Because Klim et al reported that a synthetic peptide derived from the heparin-binding domain of vitronectin supports long-term culture of hPSCs [18], we developed an Fc fusion protein containing the heparin-binding domain of vitronectin (HBD-Fc). However, HBD-Fc could not support the initial adhesion or propagation of hPSCs (data not shown). In addition, hPSCs adhered to surfaces coated with NC(D6E)-Fc mutants (Fig 1D), but almost all cells had detached from the surface within 3 days (Fig 2). The reason for the inactivity of heparin-binding domain-containing fragments could be the lower coating density of fusion proteins compared with synthetic peptides because of steric hindrance. On R-Fc- and NC-Fc-coated surfaces, hPSCs proliferated and maintained an undifferentiated state (Figs 2 and 3A and 3B). Furthermore, hiPS cells cultured on R-Fc or NC-Fc maintained the ability to differentiate into three germ layer-derived cells *in vitro* and *in vivo* (Fig 3B and 3C). Therefore, we used R-Fc and NC-Fc for differentiation studies.

To differentiate hPSCs, we determined whether hE-cad-Fc and full-length recombinant vitronectin were useful substrates for differentiation into hepatocyte—like cells. Although the use of E-cad-Fc has been reported for the differentiation of mouse ES cells into ALB-positive hepatic cells [37], this substrate could not support the differentiation of human ES and iPS cells into definitive endoderm, which is a pre-requisite for the formation of hepatocytes, because the cells detach from the E-cad-Fc substrate (Fig 4A). It has been reported that expression of E-cadherin is maintained in definitive endoderm cells differentiated from mouse ES cells [43]. However, E-cadherin expression in hPSCs diminishes during differentiation into definitive endoderm lineages presumably resulting in an inability to attach to the E-cad-Fc substrate (Fig 4D) [38]. Therefore, we conclude that hE-cad-Fc is not a suitable substrate for the initial differentiation of hPSCs to endoderm. We did find that hPSCs were able to efficiently generate definitive endoderm cells and hepatocyte-like cells on full-length recombinant vitronectin; however, a major issue with full-length vitronectin was detachment of the differentiating cells as sheets from the surface during the specification stage of the differentiation protocol (Fig 4C). A similar issue with cell detachment was observed on the NC-Fc-coated surface (Fig 5). Vitronectin is a putative target of MMP-2, and we identified increased expression of *MMP2* and *MMP14* during differentiation (Fig 4D). Therefore, cell detachment is likely attributable to cleavage of vitronectin by MMPs. Vitronectin has a predicted MMP-2 recognition site around Pro123 (EAP-APEVG), but R-Fc does not contain this sequence (Fig 1A). Therefore, we believe that hPSCs can remain adherent during differentiation into hepatic progenitor cells on the R-Fc-coated surface because they are resistant to metalloproteinase mediated cleavage.

When hiPS cells were differentiated on the R-Fc-coated surface, we detected the expression of several markers of hepatic lineage cells (Fig 6B), and differentiated cells on R-Fc showed several hepatic functions (Fig 6C and 6D). hiPSC-derived cells expressed high levels of the hepatoblast markers, *DLK1* and *AFP*. Furthermore, the gene expression profile of cells differentiated on R-Fc was similar to that of fetal liver cells (Fig 6E). Taken together, these findings suggest that the majority of differentiated cells were immature hepatoblasts, and further maturation would be needed to produce functional hepatocytes. Although many reports suggest that the differentiation of hPSCs into hepatocyte-like cells mimics the developmental process, it remains challenging to generate mature hepatocytes from pluripotent stem cells presumably because of the complex process of cell maturation that occurs during hepatogenesis. For therapeutic applications or for metabolic studies that require highly differentiated hepatocytes advances in differentiation procedures will be necessary.

In this study, we have developed a novel highly defined cell adhesion substrate, R-Fc, which is a small fragment of vitronectin containing the RGD motif. This protein can be used to both maintain the pluripotent state of hPSCs and to differentiate them into hepatocyte—like cells. Since the protein is generated as a fusion with the IgG—Fc domain, it can easily be purified from bacterial cultures use protein A affinity purification. We propose that this substrate can be used both for the maintenance of human pluripotent stem cells and in addition facilitates their differentiation to hepatic lineages. We believe that it is likely that the substrate can offer viable alternative to Matrigel in many differentiation protocols.
